# An Emerging Threat: A Systematic Review of Endocarditis Caused by Gemella Species

**DOI:** 10.7759/cureus.58802

**Published:** 2024-04-23

**Authors:** Gina N Gonzalez, Carlos D Franco, Tanya Sinha, Emilia I Ramos, Syed Faqeer Hussain Bokhari, Danyal Bakht, Maaz Amir, Muhammad Arsham Javed, Khawar Ali, Nailet Pineda Renté

**Affiliations:** 1 Internal Medicine, University College Hospital, Quito, ECU; 2 Medicine, Universidad Laica Eloy Alfaro de Manabí, Manta, ECU; 3 Medical Education, Tribhuvan University, Kathmandu, NPL; 4 Surgery, King Edward Medical University, Lahore, PAK; 5 Medicine and Surgery, King Edward Medical University, Lahore, PAK; 6 Medicine and Surgery, Mayo Hospital, Lahore, PAK; 7 General Surgery, King Edward Medical University, Lahore, PAK; 8 Orthopedic Surgery, Palmetto Hospital, Hialeah, USA; 9 Emergency Medicine, Hospital de Clinicas Manuel Quintelas, Montevideo, URY

**Keywords:** systematic review, review, tricuspid, aortic, mitral, infection, valvular heart diseases, echocardiography, gemella, endocarditis

## Abstract

Infective endocarditis caused by *Gemella* species is increasingly recognized as an emerging clinical entity. *Gemella* species are fastidious gram-positive cocci that are typically commensal organisms but can become opportunistic pathogens. This systematic review aimed to provide a comprehensive overview of endocarditis due to *Gemella* species by synthesizing existing evidence. A total of 52 case reports were identified through a rigorous search and selection process. The most prevalent causative species were *G. morbillorum* (46.3%) and *G. haemolysans* (25.9%), with a striking male predominance (79.6%). The clinical presentation was largely nonspecific, mirroring typical infective endocarditis. However, the indolent nature of the illness and fastidious growth requirements of *Gemella* species often led to diagnostic delays. Echocardiography, particularly transesophageal echocardiography, played a crucial role in the diagnosis, enabling the detection of valvular vegetation and the assessment of complications. Management posed significant challenges, including the need for broad-spectrum empirical antibiotic therapy and increasing antimicrobial resistance among *Gemella* isolates. Surgical intervention was frequently required for severe valvular dysfunction, persistent infection, or embolic complications. Despite advances in diagnosis and treatment, endocarditis due to *Gemella* species remains associated with significant morbidity and mortality, underscoring the importance of early recognition and multidisciplinary management. This review highlights the emerging clinical significance of *Gemella* species as causative agents of infective endocarditis and identifies areas for further research.

## Introduction and background

Infective endocarditis (IE) is a serious and potentially life-threatening condition characterized by microbial infection of the endocardium, most commonly affecting the heart valves [1]. Historically, IE has been predominantly associated with well-known pathogens such as Streptococcus and Staphylococcus species [2]. However, there is growing recognition of the role of fastidious bacteria in the etiology of this condition. Among these fastidious organisms, *Gemella* species have emerged as noteworthy contributors to infective endocarditis [3-7]. *Gemella *species belong to the genus *Gemella*, a group of gram-positive cocci that are facultative anaerobes. These organisms are part of the normal flora of humans [8]. While they typically exist as commensals, *Gemella* species possess virulence factors that enable them to cause disease under certain conditions. In recent years, *Gemella* species, including *G. haemolysans, G. morbillorum, and G. sanguinis*, have been increasingly implicated in cases of infective endocarditis, highlighting the importance of understanding their role in this clinical context.

Endocarditis due to *Gemella* species presents several unique challenges compared to endocarditis caused by more commonly recognized pathogens. The clinical presentation of endocarditis due to *Gemella* species often mirrors that of typical IE, with symptoms such as fever, malaise, new-onset murmurs, and signs of systemic embolization. However, the nonspecific nature of these symptoms can complicate diagnosis, leading to delays in appropriate management [9]. Furthermore,* Gemella* species are known for their fastidious growth requirements, making their isolation and identification challenging in routine microbiological cultures. The diagnosis of endocarditis due to *Gemella* species relies on a combination of clinical evaluation, echocardiography, and microbiological studies. Echocardiography, including transthoracic echocardiography (TTE) and transesophageal echocardiography (TEE), plays a crucial role in detecting valvular vegetation, assessing valvular function, and identifying complications such as abscess formation or valvular regurgitation. Microbiological confirmation of endocarditis due to *Gemella* species is often based on peripheral blood culture [9].

The management of endocarditis due to *Gemella* species poses several therapeutic challenges. Empirical antibiotic therapy must be carefully chosen to cover the spectrum of potential pathogens, including fastidious organisms like *Gemella* species. However, the increasing prevalence of antimicrobial resistance among *Gemella* isolates underscores the importance of tailored antibiotic regimens based on susceptibility testing and clinical response [10]. Surgical intervention, such as valve repair or replacement, may be necessary in cases of severe valvular dysfunction, persistent infection, or embolic complications. Despite advances in diagnostic techniques and treatment modalities, endocarditis due to *Gemella* species remains associated with significant morbidity and mortality. Therefore, there is a critical need for enhanced awareness, early recognition, and multidisciplinary management of this condition. This systematic review aims to provide a comprehensive overview of *Gemella*-associated endocarditis, including its clinical presentation, diagnostic approach, treatment strategies, and outcomes. By synthesizing existing evidence and identifying areas for further research, this review seeks to inform clinical practice, guide therapeutic decision-making, and improve patient outcomes in the management of endocarditis due to *Gemella* species.

## Review

Materials and methods

Search Strategy

This systematic review adheres to the Preferred Reporting Items for Systematic Reviews and Meta-Analyses (PRISMA) guidelines to ensure transparency and rigor in the review process. A comprehensive search of relevant literature was conducted across prominent databases renowned for their extensive coverage of medical and scientific literature, including PubMed, Embase, Web of Science, and Scopus. These databases were selected for their comprehensive collection of peer-reviewed articles, providing a robust foundation for our systematic review of endocarditis caused by *Gemella* species. The search strategy employed a meticulously curated set of keywords and phrases aligned with the objectives of the study. These included terms such as "Gemella" and "Endocarditis". Boolean operators "AND" and "OR" were strategically utilized to construct the search algorithm. For instance, the string "Gemella AND Endocarditis" focused specifically on studies addressing endocarditis caused by *Gemella* species, while the use of "OR" facilitated the inclusion of broader terms associated with the topic. To ensure the inclusion of contemporary and relevant literature, the search was limited to studies published from the inception of each database to January 2024. This timeframe allowed for the incorporation of both historical and current research, offering a comprehensive overview of endocarditis caused by *Gemella* species. Filters were applied to include studies published in the English language and those involving human subjects, in line with the objectives of our review. Additionally, manual searches of the reference lists of included studies and relevant reviews were conducted to supplement the electronic database search.

Eligibility Criteria

The eligibility criteria for this systematic review were established to ensure precision and relevance in selecting studies for inclusion. Peer-reviewed research articles, observational studies, case reports, and clinical trials were considered eligible for inclusion, reflecting a commitment to evidence-based knowledge. To maintain methodological rigor, only studies published in the English language were included, acknowledging English as the predominant language of scientific communication. The inclusion timeframe spanned from the inception of the respective databases to the present date, allowing for the synthesis of contemporary research on endocarditis caused by *Gemella* species. Conversely, exclusion criteria were carefully tailored to maintain focus and rigor. Studies not directly addressing endocarditis caused by *Gemella* species, as well as those lacking relevant outcome measures, were excluded. Non-English language publications, unpublished works, and gray literature such as conference abstracts were also excluded. Furthermore, studies presenting insufficient data on endocarditis caused by* Gemella *species were excluded to ensure the integrity of the review's findings.

Data Extraction

The data extraction process was conducted in a meticulous and structured manner to ensure the accuracy and completeness of the review findings. This process involved two stages, emphasizing thoroughness and reliability. In the initial stage, articles were screened based on the relevance indicated by titles and abstracts. Two independent reviewers assessed each article's abstract to determine its relevance to the review's focus. Articles deemed relevant or probably relevant underwent a detailed examination in the second stage. In the second stage, full-text articles meeting the inclusion criteria underwent a detailed data extraction process. Two independent reviewers utilized a standardized data extraction template within Microsoft Excel to capture and organize critical information from each study. Any discrepancies between reviewers were resolved through the adjudication of a third independent reviewer.

Results

Study Selection Process

**Figure 1:**
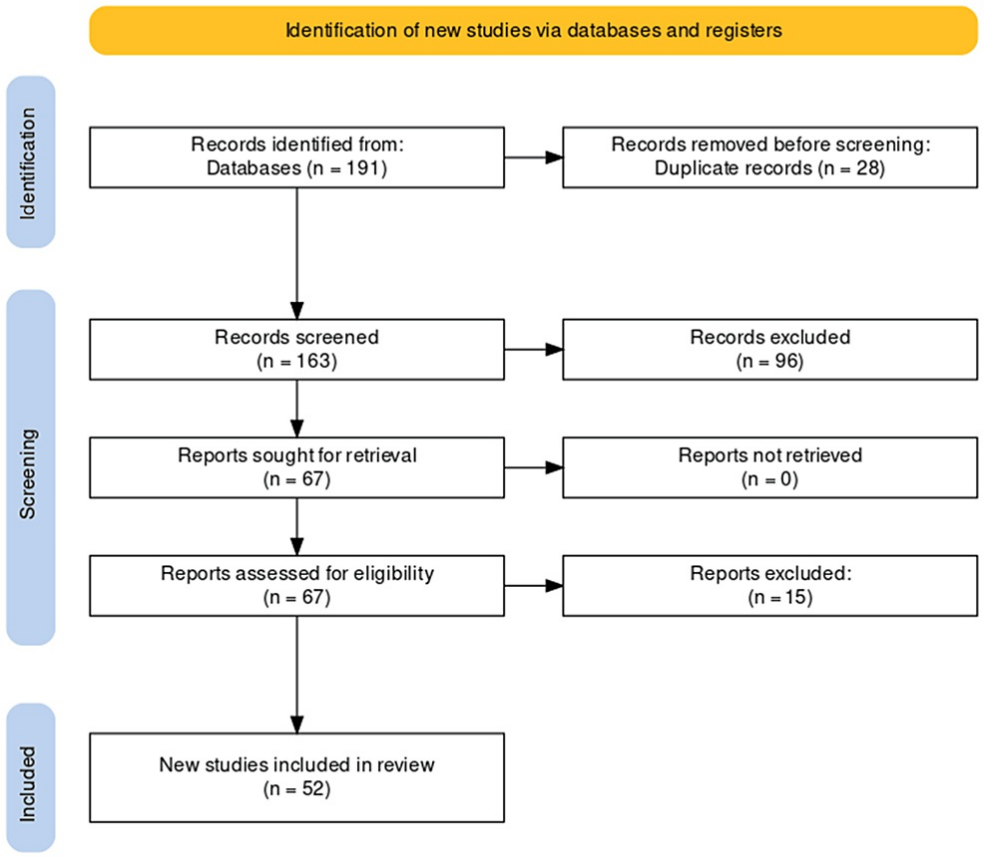
PRISMA flow diagram of selection of studies for inclusion in the systematic review.

A comprehensive search initially yielded 191 studies, from which duplicates were removed, resulting in a refined pool of 163 unique studies. Subsequent screening of titles and abstracts led to the exclusion of 96 records that did not meet predefined relevance criteria. Full-text evaluation of the remaining 67 articles resulted in the exclusion of 15 reports that did not align with stringent inclusion criteria. The culmination of this rigorous selection process identified 52 studies suitable for inclusion in the systematic review, providing a focused and robust source of evidence for the analysis of endocarditis caused by *Gemella *species. The PRISMA flowchart detailing the study selection process is presented below (Figure 1).

Study Characteristics

This systematic review included a total of 52 case reports on endocarditis caused by *Gemella *species. Among the reported cases, 43 (79.6%) were male, while 10 (18.5%) were female, indicating a male predominance. Patient ages ranged from 4 to 87 years, with a mean age of 48 years. The most prevalent causative species were *G. morbillorum* in 25 (46.3%) cases and *G. haemolysans* in 14 (25.9%) cases, followed by *G. sanguinis *in 7 (13%) cases and *G. bergeri *in 4 (74%) cases. There was one case each of infection by both *G. haemolysans *and *G. morbillorum*. In terms of valve involvement, isolated mitral and aortic valves were found to have the same incidence of involvement, with 15 (43.4%) cases each. Isolated tricuspid valve was involved in 3 (5.5%) cases. In 10 (18.5%) cases, bivalvular (aortic and mitral) involvement was observed, followed by trivalvular (mitral, pulmonary, and aortic) and panvalvular involvement in 1 case each. In prosthetic valve endocarditis, the prosthetic aortic valve (9.2%) was more commonly involved followed by the prosthetic mitral valve (1.8%). These findings underscore the diversity of *Gemella *species implicated in endocarditis and the variability in valve involvement among affected individuals (Table 1).

**Table 1:** Characteristics of the studies included in this systematic review.

Author	Year	Country	Sample size	Age	Gender	Gemella species	Valve(s) involved
Brack et al. [11]	1991	United Kingdom	1	74	Male	G. haemolysans	Mitral
Terada et al. [12]	1994	Japan	1	64	Male	G. morbillorum	Mitral and aortic
Kerr et al. [13]	1994	Ireland	1	29	Female	G. morbillorum	Mitral
Samuel et al. [14]	1995	United Kingdom	1	34	Male	G. haemolysans	Prosthetic aortic
Martin et al. [15]	1995	United Kingdom	1	75	Male	G. morbillorum	Mitral
Ukimura et al. [16]	1998	Japan	1	57	Male	*G. haemolysans*, *G. morbillorum*	Prosthetic aortic
La Scola and Raoult [17]	1998	France	3	(01) 63, (02), 74, (03) N/A	(01) Male, (02) male, (03) male	(01) *G. haemolysans*, (02) *G. morbillorum*, (03) *G. morbillorum*	(01) Mitral, (02) aortic, (03) aortic
Mosquera et al. [18]	2000	Spain	1	77	Male	G. haemolysans	Aortic
Akiyama et al. [19]	2001	Japan	1	55	Male	G. morbillorum	Aortic
Purcell et al. [20]	2001	Canada	1	12	Female	Not specified	Mitral
Zakir et al. [21]	2004	USA	1	44	Male	G. morbillorum	Prosthetic mitral
Elsayed et al. [22]	2004	Canada	1	32	Male	G. bergeri	Aortic
Zheng et al. [23]	2008	Singapore	1	67	Male	G. morbillorum	Mitral and aortic
Al Chekakie et al. [24]	2009	USA	1	44	Male	G. morbillorum	Prosthetic aortic
Godinho et al. [25]	2013	Portugal	1	72	Male	G. morbillorum	Pulmonary, mitral, tricuspid, and aortic
Ural et al. [26]	2014	Turkey	1	67	Male	G. morbillorum	Aortic
Shahani et al. [27]	2014	USA	1	73	Male	G. morbillorum	Prosthetic aortic
Yang and Tsai [28]	2014	Taiwan	1	67	Male	G. sanguinis	Aortic
Virgilio and Chieco [29]	2014	Italy	1	50	Male	G. bergeri	Aortic
Hussain et al. [30]	2014	UAE	1	24	Male	G. bergeri	Mitral and aortic
Kolhari et al. [31]	2014	India	1	34	Female	G. morbillorum	Mitral
Agrawal et al. [32]	2014	India	1	Middle-aged (exact age not mentioned)	N/A		Pulmonary
Ramchandani et al. [33]	2014	USA	1	40	Female	G. haemolysans	Prosthetic aortic
Pachirat et al. [34]	2015	Thailand	1	37	Male	G. bergeri	Tricuspid
Rosa et al. [35]	2015	Brazil	1	72	Male	G. morbillorum	Mitral
Liu et al. [36]	2016	USA	1	87	Female	G. haemolysans	Aortic
Winkler et al. [37]	2016	USA	1	67	Male	-	Mitral
Ando et al. [38]	2016	USA	1	24	Male	G. haemolysans	Mitral
M et al. [39]	2016	India	1	4	Male	G. sanguinis	Tricuspid
Shinha [40]	2017	USA	1	37	Male	G. morbillorum	Aortic
Li et al. [41]	2017	China	1	28	Male	G. morbillorum	Pulmonary
Maraki et al. [42]	2019	Greece	1	21	Male	G. sanguinis	Bicuspid aortic
Youssef et al. [43]	2019	USA	1	81	Male	G. haemolysans	Mitral
Agrawal et al. [44]	2019	USA	1	38	Male	G. haemolysans	Mitral and aortic
Emmanouilidou et al. [45]	2019	Greece	1	85	Female	G. sanguinis	Mitral and aortic
Kobayashi et al. [46]	2020	Japan	1	54	Male	G. morbillorum	Mitral
Sideris et al. [47]	2020	USA	1	53	Male	G. sanguinis	Mitral
Desai and Bonura [48]	2021	USA	1	72	Male	G. morbillorum	Mitral, pulmonary, and aortic
Singer et al. [49]	2021	Canada	1	49	Male	G. morbillorum	Aortic
Dogan et al. [50]	2021	Turkey	1	37	Male	G. morbillorum	Mitral and aortic
Patel et al. [51]	2021	USA	1	56	Female	G. morbillorum	Aortic
Tanveer et al. [52]	2021	USA	1	48	Male	G. morbillorum	Mitral
Eslinger and Ahmed [53]	2022	USA	1	63	Male	G. haemolysans	Aortic
Rabah et al. [54]	2022	USA	1	56	Male	G. haemolysans	Mitral
Shah et al. [3]	2022	USA	1	53	Male	G. sanguinis	Mitral and aortic
Filip et al. [4]	2023	Romania	1	8	Male	G. sanguinis	Mitral and aortic
Cao and Yuan [6]	2023	China	1	37	Male	G. morbillorum	Mitral and aortic
Lim et al. [55]	2023	Phillipines	1	40	Male	G. morbillorum	Mitral and aortic
Dini et al. [7]	2023	Italy	1	14	Male	G. haemolysans	Aortic
Gonzalez et al. [56]	2023	USA	1	30	Female	G. haemolysans	Tricuspid
Taimur et al. [5]	2023	Pakistan	1	31	Female	G. morbillorum	Aortic
Kassab et al. [9]	2024	USA	1	77	Female	G. haemolysans	Mitral

The main findings of the included case reports are summarized in Table 2.

**Table 2:** Summary of the studies included in this systematic review.

Author	Year	Clinical presentation	Diagnostic findings	Treatment	Outcome/prognosis
Brack et al. [11]	1991	Myalgia, headache, rigors, and night sweats	Echocardiography showed prolapse of the posterior leaflet of the mitral valve with a small mass attached suggestive of a vegetation. Blood cultures were positive for *Gemella haemolysans*	IV Benzyl penicillin and gentamicin daily. After 2 weeks, the penicillin was replaced by oral amoxicillin for 4 further weeks and the gentamicin was given IM for 2 further weeks	Following cessation of antibiotics, he continued to remain apyrexial, with negative blood cultures
Terada et al. [12]	1994	Low-grade fever and nocturnal dyspnea with a history of previous dental caries	Vegetation and rupture of chorda were observed in the anterior mitral leaflet, and vegetation in the right coronary cusps. Abnormal CBC report (Leukocytosis), elevated CRP and ESR	Treatment with penicillin G (PCG) was then started by giving IV drip injection at a daily dose of 18 million units. Diuretics and cardiotonic agents were administered simultaneously. Dental caries were treated and afterwards, GSF was administered to cope with granulocytopenia. Replacement surgery of aortic and mitral valves was performed	The patient recovered completely and was discharged on the 101st hospital day
Kerr et al. [13]	1994	Persistent lethargy, flu-like illness with night sweats and dry cough three weeks ago accompanied by swollen left knee a week ago. History of two wisdom teeth extractions three months ago	Electrocardiography showed marked left ventricular hypertrophy, and a transthoracic echocardiogram showed severe hypertrophic obstructive cardiomyopathy. No vegetations were seen. Blood cultures were positive for *Gemella morbillorum*. Abnormal CBC profile	Empirical treatment with IV benzylpenicillin and gentamicin. Toward the end of the third week of treatment, antibiotics were changed to oral erythromycin and oral rifampicin due to the development of resistance. Chlorpheniramine was given for rash resolution	Treatment continued for a total of 6 weeks after which she was discharged. She remains clinically well to date
Samuel et al. [14]	1995	Myalgia, fever, and general malaise	Blood cultures were positive for *G. haemolysans*. Transoesophageal echocardiography showed a lesion on his prosthetic aortic valve suggestive of a vegetation	IV cefuroxime and tobramycin; the latter was stopped after 14 days and oral ciprofloxacin was substituted. Cefuroxime was also stopped after another 5 days due to induced neutropenia	The patient made a good recovery and remains well with no deterioration in his prosthetic valve function
Martin et al. [15]	1995	Weight loss and lassitude of two months duration and mild ankle edema. He had a history of rheumatic fever as a child	An echocardiogram revealed mitral valve vegetations with regurgitation. Blood cultures were positive for *G. morbillorum*. Altered complete blood count profile	He was started on benzylpenicillin 1.2 g IV 4 hourly and gentamicin 80 mg IV 12 hourly. After further cultures were taken, he was given teicoplanin. Then he was given IV hydrocortisone, chlorpheniramine, and salbutamol. Then he was given rifampicin 600 mg orally 12 hourly and erythromycin 500 mg orally 6 hourly due to their sensitivity report and the side effects of previous medications	The patient received a total of four weeks of treatment and made a good recovery. He remains well six months later
Ukimura et al. [16]	1998	Abdominal pain, fever, and oliguria suggestive of renal arterial embolism.	Deranged CBC (increased WBCs and C-reactive protein). A transesophageal echocardiogram showed vegetations on the prosthetic valve during hospitalization. Blood culture was positive for Gemella species	Treated with IV ampicillin and astromycin and underwent valve replacement	The post-operative clinical course was excellent and the patient was stable
La Scola and Raoult [17]	1998	Patient 01: intermittent fever, loss of weight, and anorexia. Patient 02: intermittent fever, sweating, loss of weight, and basithoracic pain. Patient 03: intermittent fever and a weight loss of 12 kg over a period of several months	(01) Altered CBC Report. Blood cultures were positive for *G. haemolysans*. A transesophageal echocardiogram demonstrated the presence of vegetation on the mitral valve with moderate mitral valve regurgitation. (02) Deranged CBC Report. Blood cultures were positive for Gmella morbillorum. A transesophageal echocardiogram demonstrated aortic valve incompetence but failed to show any vegetation. (03) Blood cultures were positive for Gemella morbillorum. A transesophageal echocardiogram demonstrated aortic valve vegetation	(01) Treatment with amoxicillin (4 g intravenously at 6-h intervals) and amikacin (5 mg/kg of body weight intravenously at 8-h intervals) was begun. His condition improved rapidly, and after 2 weeks of this regimen, he underwent cardiac surgery to remove the motile vegetation. (02) The patient’s treatment began the day after his admission and comprised amoxicillin (4 g intravenously at 6-hour intervals) and gentamicin (1 mg/kg intravenously at 8-hour intervals). The aortic valve was successfully replaced with a prosthetic device. (03) N/A	(01) One week after surgery antibiotic therapy was discontinued. After 2 years of follow-up, he remains well. (02) Gentamicin was discontinued 1 week later, and amoxicillin was discontinued 3 weeks later. After 1 year of follow-up, he remains well. (03) N/A
Mosquera et al. [18]	2000	A 77-year-old man with a long history of hemochromatosis with chronic liver disease and arterial hypertension was admitted to the hospital because of malaise, anorexia, weight loss, and progressive dyspnea over the previous 4 months	Echocardiography revealed a vegetation on the aortic valve with moderate valvular insufficiency. *G. haemolysans* was isolated in the three blood cultures taken on admission	The patient was treated with penicillin G (24 MU IV daily, divided into six doses) and tobramycin (100 mg twice a day IV) for 2 weeks	The patient showed improvement in his clinical status as well as sterilization of the blood cultures. In a follow-up visit 30 days after discharge, the echocardiogram did not show any vegetation
Akiyama et al. [19]	2001	A 55-year-old man with persistent fever and nocturnal dyspnea was referred to hospital. He had a history of noninsulin-dependent diabetes mellitus and type B hepatitis	Echocardiography demonstrated massive aortic regurgitation with a vegetation-like high-density echo on the right coronary cusp and moderate mitral regurgitation, but the tricuspid and pulmonary valves did not show any abnormality	The patient was treated surgically. Postoperatively, the patient was given IV tobramycin (120 mg/day), cefmetazole sodium (3 g/day), and fosfomycin (3 g/day) for a 6-week period, which resulted in a bacteriologic cure	After medical therapy, the patient remains well without fever or cardiac symptoms and is followed up as an outpatient
Purcell et al. [20]	2001	A 12-year-old girl with congenital heart disease (mitral stenosis, ventricular septal defect, and patent ductus arteriosus) repaired at six months of age presented to her community pediatrician with a cough, decreased appetite, and a two-week history of fatigue	An echocardiogram revealed marked mitral insufficiency with flail posterior mitral leaflet, thickened mitral leaflets with vegetations, and an enlarged left atrium and ventricle. A diagnosis of infective endocarditis and ruptured chordae tendinae of the posterior mitral leaflet was made	The patient was started with vancomycin. Gentamicin 70 mg given intravenously every 8 h was added on the third day for synergism after consultation with the infectious diseases service. Twelve days after the initial diagnosis, when the organism was identified as a Gemella species sensitive to penicillin, the vancomycin was discontinued, and penicillin 2,200,000 IU was given intravenously every 6 h	The patient's hospital course was uneventful. She remained afebrile and her fatigue resolved. She was discharged home after two weeks in hospital on six weeks of penicillin (2,200,000 IU intravenously every 6 h) and gentamicin (70 mg intravenously every 8 h). At an eight-week follow-up visit with the IWK Grace Health Centre (Halifax, Nova Scotia), she was doing well with normal strength and activity levels
Zakir et al. [21]	2004	A 44-year-old human immunodeficiency virus-positive man with a history of active IV drug use and a mitral valve replacement only 9 months earlier for native-valve endocarditis due to Staphylococcus aureus was admitted complaining of pleuritic chest pain, shortness of breath, fever, and chills	A TTR revealed severe regurgitation through incompetent leaflets of the bioprosthetic mitral valve. The leaflets of the mitral bioprosthesis were thickened and studded with globular echo densities consistent with vegetations	Ceftriaxone for 4 weeks, with gentamicin added during the first 2 weeks of therapy	The patient was stable 6 months after discharge although he received no surgical therapy
Elsayed et al. [22]	2004	A 32-year-old Western European man visiting Canada, with a known history of asthma, hypercholesterolemia, and gastroesophageal reflux disease, presented at the hospital with a 1-month history of intermittent low-grade fever, chills, diaphoresis, fatigue, myalgia, lightheadedness, and anorexia and a 1-week history of worsening dyspnea and discomfort on the left side of his chest	A transesophageal echocardiogram was performed, demonstrating the presence of severe aortic regurgitation on a bicuspid valve, an aortic valve ring abscess, and a torn noncoronary cusp harboring multiple vegetations	Ampicillin and gentamicin. Twenty-four hours later, the patient underwent ring abscess curettage and replacement of the aortic valve with a mechanical prosthesis	The patient was discharged from the hospital and sent home on antibiotics. The planned duration of antibiotic therapy was 4 to 6 weeks. Before discharge, he had developed a second-degree heart block requiring temporary pacing
Zheng et al. [23]	2008	Shortness of breath associated with chills and rigors for three days with a history of left lobar pneumonia, end-stage renal disease mild to moderate aortic regurgitation, and treated syphilis	TTE showed concentric left ventricular hypertrophy, Multiple vegetations on aortic and mitral valves with thickening of the mitral valve, and culture positive for *G. morbillorum*	Ampicillin and gentamycin administration and surgery were planned	He died due to acute myocardial infarction from worsening aortic incompetence before the surgery
Al Chekakie et al. [24]	2009	Increased shortness of breath and decreased urine output for the past 4 days and a history of bicuspid aortic valve replacement	A TTE showed a stable aortic prosthetic valve with mild aortic regurgitation, mild mitral regurgitation, and a left ventricular ejection fraction of 15%, Cultures obtained from the aortic valve grew *G. morbillorum*	Vancomycin, gentamicin, and rifampin later changed to ampicillin and gentamicin, aortic valve replacement with a 22-mm homograft, LVAD was placed	The patient progressively worsened and developed multiorgan failure. He exsanguinated (presumably from a left ventricular assist device cannulation site) and died on postoperative day 14
Godinho et al. [25]	2013	Fever, abdominal pain, long-standing dyspnea on exertion, and petechiae on lower limbs	Thickening of all four valves with vegetations on mitral and tricuspid valves, isolation of *G. morbillorum* in three different blood cultures	Antibiotic therapy with vancomycin and gentamycin, mitral and aortic valve replacement surgery with biological prostheses, and also tricuspid valve annuloplasty	He was discharged from the hospital and continues to do well
Ural et al. [26]	2014	Fever, fatigue, deterioration of the overall status	Decreased hemoglobin, elevated ESR, CRP, and procalcitonin. Increased spleen size at USG and CT abdomen. Left occipital lobe infarction on cerebral MRI. TTE revealed aortic valve vegetation. *G. morbillorum* identified on hemocultures	Treatment with ampicillin-sulbactam and gentamicin was started, which was then replaced by a broader-spectrum meropenem and vancomycin	The patient denied early surgical treatment. After 4 weeks of antibiotic therapy, he developed left hemiplegia. Was then referred to an external site for operation by his own will for valve surgery
Shahani et al. [27]	2014	Fever, chills, fatigue, and bilateral lower extremity swelling with a history of coronary artery bypass graft with an aortic valve replacement for aortic stenosis. Dental caries in bilateral lower molars were also found	Leukocytosis, anemia, and elevated CRP. TTE demonstrated a significant decrease in the ejection fraction along with a mobile vegetation attached to the bioprosthetic aortic valve associated with a perivalvular abscess. Blood culture and later gene sequencing revealed *G. morbillorum*	6-week regimen with penicillin G along with gentamicin for the first 2 weeks. Dental extractions were performed to minimize the risk of reinfection. Subsequently, cardiac surgery was conducted, involving debridement of the perivalvular abscess and bioprosthetic aortic valve replacement	The patient completed his 6-week regimen of IV antibiotics and had a successful outcome with his infective endocarditis
Yang and Tsai [28]	2014	Fever, chills, and generalized weakness and malaise with a history of rheumatic heart disease and gouty arthritis	Leukocytosis, anemia, elevated CRP, fibrinogen and D-dimer, and hypoalbuminemia were found. TTE showed aortic stenosis and aortic regurgitation with suspected paravalvular abscess. Mild mitral regurgitation, atrial fibrillation, and small pericardial effusion were noted. Blood cultures positive for *G. sanguinis*	Treatment was started with moxifloxacin and ceftriaxone. Switched after 1 day to vancomycin and gentamicin. Shortly thereafter, was adjusted to penicillin and the surgeon was consulted. The patient subsequently underwent surgery with a successful aortic prosthetic valve replacement	He continued to receive IV penicillin treatment for 6 weeks and was discharged with a stable condition. No evidence of recurrence after 3 months of evaluation
Virgilio and Chieco [29]	2014	Fever, shivering, refractoriness to common oral antibiotics, and past history of bicuspid aortic valve	Leukocytosis, elevated CRP, and ESR. TTE revealed mobile vegetation attached to the anterior cusp of the aortic valve. Blood cultures positive for *G. bergeri*	Commenced on IV amoxicillin-clavulanate associated with amikacin. Therapy switched to ceftriaxone plus gentamicin according to antibiogram results	The patient became apyrexial on this antibiotic therapy, which was prescribed for 1 month
Hussain et al. [30]	2014	Headache and left hemiparesis, with a past history of anemia	Leukocytosis, anemia, elevated platelet count, CRP, and serum procalcitonin. Hematuria was also seen. CT brain revealed hypodensities in the right parietal cortex and left posterior frontal cortex, indicating ischemic infarcts. The echocardiogram showed thickened aortic cusps with vegetations causing severe aortic regurgitation. The anterior leaflet of the mitral valve thickened with multiple vegetations and mild mitral regurgitation. The left atrium and left ventricle dilated. Blood cultures positive for *G. bergeri*	Started empirically on IV ceftriaxone and gentamicin. Later, GCS dropped to 8/15 and CT revealed right fronto-temporoparietal intra-axial hematoma for which right-sided decompression craniotomy and insertion of intracerebral pressure monitoring catheter was done. He then received all brain protective measures as per ICU protocol for high ICP. Based on the susceptibility test of the organism, the same antibiotics were continued	ICP remained persistently high despite brain protective measures. A follow-up CT scan revealed a large hemorrhagic infarct in the right MCA territory with brain swelling and mass effect. The patient remained hypotensive, needing full inotropic support. Lost brain stem reflexes and deceased after 20 days
Kolhari et al. [31]	2014	low-grade fever, swelling of lower limbs, facial puffiness, breathlessness, tachycardia, Pallor, pedal edema, elevated jugular venous pulse, hyperdynamic apex and ejection systolic murmur, basal crepitations and a palpable spleen	ECG showed major left ventricular hypertrophy and abnormal repolarization in the lateral ECG leads. Two-dimensional echocardiography showed localized septal hypertrophy (2.2 cm). There was a large (22×12 mm), mobile vegetation on the anterior mitral leaflet, and mitral regurgitation (MR) was quantified as mild. Mild pericardial effusion was also seen	IV crystalline penicillin and levofloxacin and mitral valve replacement	Postoperatively the patient improved symptomatically and was discharged after completion of 6 weeks of parenteral antibiotic treatment. At 6 months of follow-up, the patient is asymptomatic
Agrawal et al. [32]	2014	progressively worsening exertional fatigue and dyspnea; a history of being treated for infective endocarditis by *G. morbillorum*	severe pulmonary regurgitation, the deformed valve appeared echocardiographically convoluted like a ‘cauliflower’	This patient underwent successful replacement of the pulmonary valve with a bioprosthetic valve and patch closure of the ASD	The patient has been stable and asymptomatic after 6 months of follow-up
Ramchandani et al. [33]	2014	progressive shortness of breath, lower extremity edema, fevers with temperatures to 39°C, myalgias and night sweats, and a history of congenital aortic coarctation, repaired at age 4, and a bicuspid aortic valve with subsequent symptomatic aortic stenosis requiring bioprosthetic aortic valve replacement and root enlargement at age 32	a transthoracic echocardiogram was suggestive of endocarditis with a paravalvular aortic root abscess, 16s rRNA PCR identified *G. haemolysans* in all tissue specimens. Pathology of her explanted heart showed both ischemic and embolic infarcts of varying ages	vancomycin and ceftriaxone later changed to ampicillin and gentamycin, extensive debridement mandating reconstruction with a TAH, and then a heart transplant 6 months later	She continues to do well seven months afterward
Pachirat et al. [34]	2015	Prolonged fever, weight loss, dyspnea on exertion	TTE revealed tricuspid valve vegetation with moderate regurgitation. Routine blood cultures were negative, but PCR/sequencing of valve tissue identified *G. bergeri*	Tricuspid valve repair and vegetectomy	Discharged with good outcome after one month
Rosa et al. [35]	2015	Dry cough, fever, anorexia, loss of body weight, dyspnea, hypotension, tachycardia, fever, oliguria and signs of poor peripheral perfusion	CXR showed pulmonary congestion and cardiomegaly. CBC showed leukocytosis and normocytic anemia. Elevated ESR and CRP were seen. TEE revealed a thickened mitral valve, anterior leaflet flail, and regurgitation. Lung ultrasound showed diffuse bilateral B-lines. *G. morbillorum *isolated in blood cultures	Initial empirical antimicrobial therapy with ceftriaxone plus gentamicin. Hemodynamic instability treated with norepinephrine and intra-aortic balloon pump counterpulsation implant. Emergency mitral valve replacement surgery was done. After antimicrobial susceptibility tests, the regimen was changed to penicillin G plus gentamicin	Bioprosthetic mitral valve replacement led to improved cardiogenic shock, followed by recurrence on Day 27, requiring mechanical ventilation. Emergency valve replacement was attempted again, but the patient died during the procedure
Liu et al. [36]	2016	Back pain, weight gain, jugular venous distention, rales, edema in lower extremities	TTE showed vegetation on non-coronary aortic leaflet and mild aortic stenosis. Blood cultures grew *G. haemolysans*	Treated with ampicillin and gentamicin	The patient died unexpectedly one month later
Winkler et al. [37]	2016	Acute chest pain, nausea, and diaphoresis, suggesting embolic acute ST-segment-elevation myocardial infarction	The electrocardiogram showed a right bundle branch block with anterolateral ST-segment elevations and reciprocal inferior ST-segment depressions. TTE revealed mitral valve vegetation	Milrinone, diuretics, aspirin, clopidogrel, heparin	The patient died within 2 days due to severe heart failure and septic shock secondary to a central-line infection
Ando et al. [38]	2016	Malaise, headache, fever, rigors after treatment for parotitis and *G. haemolysans* bacteremia	Elevated white cell count, chest radiograph showed enlarged cardiac silhouette. Echocardiography revealed aortic and mitral valve vegetations with severe regurgitation	Urgent mechanical aortic valve replacement and mitral valve repair, IV penicillin G, and vancomycin	Prolonged hospitalization, discharged with lifelong warfarin and IV ceftriaxone for 7 weeks
M et al. [39]	2016	High-grade fever, chills, rigor, cough, weight loss, abdominal distension	Echocardiography showed tricuspid valve vegetation with mild regurgitation. Gram-positive cocci in pairs identified as *G. sanguinis*	Initially ceftriaxone and amikacin, then vancomycin and gentamicin for 6 weeks based on susceptibility testing	Improved and discharged after 8 weeks with regular follow-up
Shinha [40]	2017	Fever, chills, fatigue in a patient with IV drug abuse history	Systolic ejection murmur at apex. TTE revealed aortic valve vegetation. Multiple blood cultures positive for Gram-negative coccus	Not mentioned	Not mentioned
Li et al. [41]	2017	Fever, shortness of breath, exertional fatigue, dyspnea, weight loss for 3 months	TTE showed pulmonary valve vegetation with moderate regurgitation, ventricular septal defect, atrial septal defect, and double-chambered right ventricle. Blood cultures grew G. morbillorum	Aggressive antibiotic therapy followed by pulmonary valve replacement with aortic bioprosthetic valve, repair of ventricular and atrial septal defects, reconstruction of the right ventricular outflow tract, and excision of vegetations	Uneventful postoperative recovery, asymptomatic at 3-month follow-up
Maraki et al. [42]	2019	Fever, poor appetite, weakness, fatigue, marked weight loss, arthralgias after the dental procedure	TTE and TEE revealed a bicuspid aortic valve with vegetation on the posterior leaflet, moderate to severe aortic regurgitation	Empiric IV ceftriaxone, gentamicin, and daptomycin initially, then ceftriaxone for 6 weeks and gentamicin for 2 weeks. Aortic valve replacement after 6 weeks	Successful treatment with valve replacement
Youssef et al. [43]	2019	Dyspnea, weakness, exertional dyspnea	New systolic murmur, echocardiography showed mitral valve vegetation with moderate regurgitation. Blood cultures grew *G. haemolysans*	Initially vancomycin and ceftriaxone, then penicillin and gentamicin. Advised surgical mitral valve replacement but opted for conservative management	Developed cardiogenic shock and severe pulmonary edema, passed away
Agrawal et al. [44]	2019	Chronic mastoiditis with exertional dyspnea for 1 month	TTE showed aortic regurgitation and aortic valve abscess. TEE revealed a bicuspid aortic valve with vegetations. Blood cultures grew *G. haemolysans*	IV ampicillin and gentamicin followed by mechanical aortic valve replacement and bovine reconstruction of the left ventricular outflow tract	Discharged after 3 weeks of IV antibiotics after negative blood cultures. Removal of periapical abscess identified as the source of infection
Emmanouilidou et al. [45]	2019	Fever, right hemiparesis, and strabismus	TTE showed mitral valve vegetation. A brain CT scan revealed ischemic damage. Blood culture positive for *G. sanguinis*	Empiric treatment with ceftriaxone, clindamycin, and acetylsalicylic acid followed by vancomycin and gentamicin	Discharged after 6 weeks of antibiotic therapy with no definite vegetation or perivalvular abscess
Kobayashi et al. [46]	2020	Fever, leg edema, and dyspnea	Roth spots, Janeway lesions, Osler nodules, and purpura on lower limbs. Infective endocarditis and infection-related pauci-immune necrotizing crescentic glomerulonephritis. Blood culture positive for *G. morbillorum*	Initial treatment with penicillin G and gentamicin, followed by ceftriaxone, daptomycin, and vancomycin. Steroid pulse therapy was administered	Surgical mitral valve replacement performed after recurrent bacteremia. Complete resolution of glomerulonephritis post-steroid therapy
Sideris et al. [47]	2020	Malaise, thigh and finger pain, and new pansystolic murmur	TTE and TEE revealed severe mitral insufficiency with vegetations. Blood culture positive for *G. sanguinis*	Empirical treatment with vancomycin and ceftriaxone followed by mechanical mitral valve replacement	Successful recovery post-operative, with office follow-up showing appropriate recovery
Desai and Bonura [48]	2021	Chronic anemia, worsening renal function, and unintentional weight loss	Renal dysfunction, severe anemia, elevated inflammatory markers, and atrial fibrillation. Imaging was initially negative, but later abdominal imaging identified splenic infarcts prompting suspicion of possible cardiac vegetation on re-evaluated echocardiogram. Blood culture positive for *G. morbillorum*	Empirical treatment with vancomycin, then cefazolin. Valve replacement surgery was deferred initially due to high surgical risk	Discharged after 6-week antibiotic course with marked clinical improvement. A follow-up echocardiogram showed no need for surgical correction
Singer et al. [49]	2021	Orthopnea, dyspnea, and weight loss	TEE revealed vegetation on the aortic and mitral valves. Blood culture positive for *G. morbillorum*	Empirical treatment with piperacillin-tazobactam, then ceftriaxone and gentamicin	Discharged on ceftriaxone with full recovery at 2-year follow-up.
Dogan et al. [50]	2021	Fever, weight loss, and right upper quadrant pain	TTE revealed vegetations on the bicuspid aortic and mitral valves. Blood culture positive for *G. morbillorum*	Initial treatment with ceftriaxone. Valve replacement surgery was performed due to persistent vegetation	Successful medical-surgical management with complete recovery
Patel et al. [51]	2021	Fevers, abdominal pain, and diarrhea	TTE showed aortic valve thickening. Blood culture positive for *Actinomyces odontolyticus* and *G. morbillorum*	Empirical treatment with ceftriaxone and metronidazole. Discharged on ceftriaxone and apixaban	Resolution of symptoms post-antibiotic therapy. Discharged for a dental follow-up
Tanveer et al. [52]	2021	Weight loss, fatigue, night sweats, and shortness of breath	An echocardiogram revealed mitral valve vegetations. CT showed splenomegaly with old infarcts. Blood cultures identified *G. morbillorum*	Vancomycin and ceftriaxone initially, followed by ceftriaxone alone post-mitral valve replacement	Improvement in symptoms, negative blood cultures post-therapy
Eslinger and Ahmed [53]	2022	Poor dental hygiene with fatigue, malaise, and acute dyspnea	TTE is consistent with aortic valve vegetation. Panorex and TTE revealed no dental abscess. Blood cultures positive for *G. haemolysans*	Vancomycin and cefepime, then penicillin G with gentamycin for 4 weeks	Cleared infection, required intermittent transfusions post-discharge
Rabah et al. [54]	2022	History of root canal presented with low-grade fever	TEE showed mitral valve vegetation. Blood cultures positive for *G. haemolysans*	Vancomycin and ceftriaxone initially. Scheduled for mitral valve replacement post-treatment completion	Successful treatment without complications
Shah et al. [3]	2022	Chest pain and palpitations	TEE revealed severe aortic insufficiency and MR	Urgent double valve replacement surgery (aortic and mitral valves)	The patient died due to complications post-surgery.
Filip et al. [4]	2023	Abdominal pain and fever	Admission echocardiography showed paravalvular aortic abscess and mitral valve vegetation	IV antibiotics followed by complex surgical intervention (Ross operation and coarctectomy)	Improved condition post-surgery, discharged in good clinical state
Cao and Yuan [6]	2023	Intermittent fever and aortic valve malformation	TTE indicated aortic valve vegetation. Blood cultures grew *G. morbillorum*	Empirical antibiotic therapy with ceftriaxone and netilmicin	Stable condition upon discharge for further cardiac surgery
Lim et al. [55]	2023	Left leg weakness and diastolic murmur	TTE and TEE revealed aortic and mitral valve vegetations. MRI showed cerebral infarct	Empirical antibiotics followed by open-heart surgery for valve replacement	Discharged to a rehabilitation center post-surgery
Dini et al. [7]	2023	Congenital aortic stenosis with fever	TEE revealed a pseudoaneurysm of mitral-aortic intervalvular fibrosa. Blood cultures positive for *G. haemolysans*	IV antibiotics followed by aortic valve replacement	Symptom-free post-recovery
Gonzalez et al. [56]	2023	Hepatitis C and IV drugs with weakness and fever	TTE showed tricuspid valve vegetation. Blood cultures positive for *P. aeruginosa* and *G. haemolysans*	Urgent tricuspid valve replacement with pacemaker implantation	Improved cardiac function post-surgery
Taimur et al. [5]	2023	Fever, exertional dyspnea, myalgias, edema, and anorexia	TTE showed aortic valve regurgitation, mild to moderate MR, left ventricular diastolic dysfunction grade II, and tricuspid regurgitation	Empirical treatment with ceftriaxone and gentamycin, based on sensitivity results	Responded well to medical treatment, evidenced by reduced vegetation size and symptom improvement; no surgical intervention required
Kassab et al. [9]	2024	End-stage renal disease, paroxysmal atrial fibrillation, and recent shoulder injury with malaise, dizziness, poor appetite, fever, and leukocytosis	TTE showed mildly thickened mitral valve leaflets, and trace MR. TEE showed large mobile vegetation on the anterior mitral leaflet. MRI revealed septic emboli with acute infarcts. Blood cultures positive for *G. haemolysans*	Empirical treatment with IV cefepime and vancomycin later switched to IV ceftriaxone based on the sensitivity	The patient's encephalopathy worsened, not suitable for surgery; transitioned to comfort measures and deceased shortly after

Discussion

This systematic review aimed to provide a comprehensive overview of infective endocarditis caused by* Gemella* species, a group of fastidious gram-positive cocci that are typically commensal organisms but can be opportunistic pathogens. The findings of this review underscore the emerging recognition of *Gemella* species as causative agents of endocarditis, a condition that has been historically associated with more commonly known pathogens. The review identified 52 case reports of endocarditis due to *Gemella* species, highlighting the diversity of *Gemella* species implicated, with *G. morbillorum* (46.3%) and *G. haemolysans* (25.9%) being the most prevalent species. The male predominance (79.6%) observed in these cases aligns with the general epidemiological pattern of infective endocarditis, although the underlying reasons for this predilection warrant further investigation [57,58].

The clinical presentation of endocarditis due to *Gemella* species varies widely, ranging from nonspecific symptoms such as fever, malaise, and fatigue to more specific manifestations like myalgia, dyspnea, and focal neurological deficits [3,5,9,30,45]. Additionally, predisposing factors such as dental procedures, congenital heart disease, and immunocompromised states were frequently noted among the patients [5,12,27,42]. However, the indolent and insidious nature of the illness, coupled with the fastidious growth requirements of *Gemella* species, often led to delays in diagnosis and appropriate management [9]. This underscores the importance of maintaining a high index of suspicion, particularly in patients with predisposing risk factors or persistent unexplained clinical manifestations. Diagnostic modalities, primarily echocardiography and blood cultures, played crucial roles in confirming the diagnosis of endocarditis and identifying the causative organism. Echocardiography, particularly TEE, enabled the detection of valvular vegetations, assessment of valvular function, and identification of complications. Blood cultures were instrumental in isolating *Gemella *species, facilitating targeted antibiotic therapy. The review revealed a wide range of valve involvement, with both native and prosthetic valves being affected, further emphasizing the need for thorough evaluation in suspected cases.

The management of endocarditis due to *Gemella *species posed several challenges. Empirical antibiotic regimens typically included broad-spectrum agents such as penicillin and gentamicin followed by tailored regimens based on susceptibility testing and clinical response [4,45,48,53]. The review highlighted the increasing prevalence of antimicrobial resistance among *Gemella* isolates, underscoring the importance of judicious antibiotic use and the potential need for combination therapy or alternative treatment strategies. Surgical intervention, including valve replacement or repair, was pursued in cases of severe valvular damage, persistent infection, or complications such as abscess formation and embolic events [3,50]. Overall outcomes were generally favorable, with most patients showing clinical improvement and resolution of infection following appropriate medical and/or surgical management. However, a small proportion of cases experienced complications or succumbed to the illness, underscoring the importance of timely diagnosis and comprehensive treatment.

This systematic review has several strengths, including the comprehensive search strategy, rigorous study selection process, and detailed data extraction. However, it is essential to acknowledge the limitations inherent in the inclusion of case reports, which may introduce potential selection bias and limit the generalizability of the findings. Additionally, the heterogeneity in reporting and variability in clinical management approaches across the included cases may have influenced the interpretation of the results.

## Conclusions

This systematic review highlights the emerging role of *Gemella* species as causative agents of infective endocarditis, a condition with significant morbidity and mortality. The findings underscore the importance of maintaining a high index of suspicion, particularly in patients with predisposing risk factors or persistent, unexplained clinical manifestations. Prompt diagnosis, tailored antimicrobial therapy, and timely surgical intervention, when indicated, are crucial for optimal patient outcomes. Future research should focus on elucidating the virulence mechanisms of *Gemella* species, identifying risk factors for infection, and developing evidence-based guidelines for the management of endocarditis due to *Gemella* species.
